# The Effect of Intrahippocampal Insulin Infusion on Spatial Cognitive Function and Markers of Neuroinflammation in Diet-induced Obesity

**DOI:** 10.3389/fendo.2018.00752

**Published:** 2018-12-11

**Authors:** Joanne M. Gladding, Kirsten N. Abbott, Christopher P. Antoniadis, Angela Stuart, Denovan P. Begg

**Affiliations:** ^1^Department of Behavioural Neuroscience, School of Psychology, UNSW Sydney, Sydney, NSW, Australia; ^2^Department of Medicine, School of Medicine, Griffith University, Gold Coast, QLD, Australia; ^3^Department of Pharmacology, School of Medicine, UNSW Sydney, Sydney, NSW, Australia

**Keywords:** insulin, hippocampus, spatial cognition, high fat diet, diet induced obesity

## Abstract

Obesity and high fat diet consumption contribute to the development of metabolic disorders, insulin resistance, neuroinflammation, and cognitive impairments. CNS administration of insulin into the brain can attenuate these cognitive impairments. The present study investigated whether hippocampal-dependent spatial memory impairments in a dietary induced mouse model of obesity could be improved by the direct administration of insulin into the hippocampus and whether this was associated with markers of hippocampal inflammation. C57Bl/6J mice consumed a low fat or high fat diet for 16 weeks and continuous intrahippocampal saline or insulin infusion for the final 4 weeks, during a period of behavioral testing, before gene expression analysis was performed. The high fat diet group demonstrated poorer spatial memory performance in the Morris water maze and Y-maze, supporting the hypothesis that high fat diet leads to hippocampal dependent cognitive impairment. Insulin infusion into the hippocampus reversed the deficit of high fat diet consumption on both of the tasks. Increased expression of inflammatory markers was detected in the hippocampus in the high fat diet group and expression of these markers was ameliorated in insulin infused mice. This demonstrates that CNS insulin can improve hippocampal-dependent memory and that hippocampal inflammation may be a factor in the development of cognitive deficits associated with diet-induced obesity. Furthermore, these data suggest that insulin may act to attenuate high fat diet induced cognitive deficits by reducing neuroinflammation.

## Introduction

The increase in obesity rates over the past 40 years has occurred so rapidly that the problem is commonly referred to as a global epidemic (1, 2). Current projections are that approximately one-fifth of adults world-wide are likely to be obese by 2025 (3), rendering increasing obesity a serious public health concern. The chronic consumption of diets high in saturated fats directly contributes to the development of obesity by promoting positive energy balance and subsequent weight gain (4–6). However, in addition to contributing to obesity, high fat diet (HFD) consumption is increasingly associated with impairments in cognition and memory (7–12). Learning and memory dependent on the hippocampus appears to be particularly susceptible to HFD-induced damage (7, 8, 11, 13). A growing body of literature demonstrates impaired performance on hippocampal-dependent memory tasks in animals consuming a HFD (7, 8, 11, 13), including the Morris Water Maze (8), T-maze (14), and object location memory tasks (11). While the specific mediator of HFD-induced impairment on hippocampal-dependent tasks remains unknown, a growing number of studies provide evidence for a role of neuroinflammation (7, 11, 13, 15) and disruptions in central insulin signaling (14, 16, 17), with the hippocampus having a high density of insulin receptors (18, 19).

Animals consuming a HFD have higher concentrations of proinflammatory cytokines within the hippocampus, including interleukin-1-beta (IL-1β), interleukin-6 (IL-6), and tumor necrosis factor-α (TNF-α), than animals consuming standard chow (7, 15, 20–22). These increased concentrations of cytokines are associated with HFD-induced impairment in objection location recognition memory (21) and spatial learning (15, 20). Ongoing consumption of HFD contributes to a state of chronic low-grade inflammation in the periphery, which is argued to contribute to the onset of systemic insulin resistance and disrupted glucose metabolism (23). A similar relationship is proposed to exist within the central nervous system (CNS) (15, 20, 24), which is normally insulin sensitive but can become insulin-resistant. Systemic insulin resistance induced in mice as a result of HFD reduces insulin receptor genes, insulin sensitive glucose transporter proteins, and activation of downstream effectors of insulin signaling pathways, such as phosphatidylinositol-3-kinase (PI3K) and Akt, in the hippocampus (14, 17, 25). The cause of brain insulin resistance may be linked to the release of proinflammatory cytokines.

Circulating free fatty acids from excessive HFD consumption initiates microglial proliferation in the CNS (26), leading to an increase in immune cell infiltration and the production of proinflammatory mediators (24). In support of the role that insulin signaling plays in cognition, intrahippocampal administration of insulin has been shown to improve performance on simple learning tasks (27), the Morris Water Maze (28, 29), and spontaneous alternation in spatial memory maze tasks (25). It was recently demonstrated that intracerebroventricular (ICV) insulin administration improves spatial memory performance and decreases the associated hippocampal neuroinflammation (30).

The present study aimed to further investigate the relationship between HFD consumption, hippocampal neuroinflammation, insulin signaling, and impaired hippocampal-dependent cognition. This study used a diet-induced obesity (DIO) model to determine the effects of chronic intrahippocampal insulin administration on hippocampal-dependent cognitive impairments in HFD fed C57BL/6J mice in the Morris Water Maze and Y-maze. To determine the effects of insulin administration on hippocampal neuroinflammation the mRNA of markers of inflammation were assessed as were the growth factors brain-derived neurotrophic factor (BDNF) and insulin-like growth factor 1 (IGF-1).

## Materials and Methods

### Animals

Ninety two male C57BL/6J mice were obtained at 8 weeks of age from Animal Resources Center (Canning Vale, WA) in two cohorts (*n* = 60 and *n* = 32). Mice were housed 4–5 per cage (73 × 23 × 14 cm) under climate-controlled conditions (22 ± 2°C) with a 12 h light-dark cycle (lights on 0700 h). Mice were allowed 1 week to acclimate to housing conditions, during which time they were provided *ad libitum* access to standard laboratory chow and tap water. Mice were handled and weighed upon arrival to allocate groups to maintain equal starting weights for groups. All experiments were performed in accordance with the guidelines of the Australian Code of Practice for the Care and Use of Animals for Scientific Purposes after approval by the University of New South Wales Animal Care and Ethics Committee.

At the completion of the study (16 weeks of diet) mice were euthanized by intraperitoneal injection of sodium pentobarbital (60 mg/kg; Virbac, Milperra, NSW) and the hippocampus was dissected and snap frozen in liquid nitrogen. It was then stored at −80°C for subsequent analysis of gene expression by RT-PCR. Peripheral tissues including epididymal fat, liver, extensor digitorum longus muscle and kidney were manually weighed.

### Diet and Surgery

Cages of mice were randomly allocated to low fat diet (LFD, Specialty feeds, Canningvale, WA; *n* = 46) or high fat diet (HFD, Specialty feeds; *n* = 46) for 12-weeks. The LFD contained 7% fat w/w and the HFD contained 21% fat w/w, composed from safflower oil (1.5 g/100 g) and clarified butter/ghee (5.5 g/100 g and 19.5 g/100 g, respectively). Apart from fat and carbohydrate content diets had identical compositions, where a portion of the fat (ghee) was replaced by carbohydrate (wheat starch) in the LFD, see Table 1. Diets were based on the American Institute of Nutrition Guidelines (AIN93) and were therefore nutritionally complete. Mice were maintained on their respective diets until time of sacrifice. Body weight and food intake was measured throughout the experiment.

**Table 1:** Nutritional Compositions of the LFD and HFD from Specialty Feeds Diets^*^.

	**Calculated Nutritional Parameters (g/lOOg %**)	
	**LFD**	**HFD**
Protein (Casein Acid)	19.5	19.5
Total fat	7	21
Clarified butter (ghee)	*5.5*	*19.5*
High linoleic safflower oil	*1.5*	*1.5*
Total carbohydrates	68.5	54.5
Sucrose	*34.1*	*34.1*
Cellulose	*5*	*5*
Wheat starch	*27.2*	*13.2*
Dextrinized starch	*2.2*	*2.2*
Crude fibre	4.7	4.7
AD fiber	4.7	4.7
%Total energy from lipids	15.7%	40%
		**Other ingredients**	
DL Methionine	0.3	0.3
Calcium carbonate	1.7	1.7
Sodium chloride	0.26	0.26
AIN93 Trace Minerals	0.14	0.14
Potassium citrate	0.26	0.26
Potassium dihydrogen phosphate	0.69	0.69
Potassium sulfate	0.16	0.16
Choline chloride (75%)	0.25	0.25
AIN93 Vitamins	1	1
USP Cholestrol	0.15	0.15
Oxicap E2	Trace	Trace

* *Diets obtained from Specialty Feeds (Canmng Vale, WA). Italic values indicate the individual components of the total fat and total carbohydrate diet contents*.

### Surgical Procedure for Drug Infusion

At the end of the 12 weeks of diet, animals were allocated to receive insulin or saline infusion and underwent surgery for bilateral cannulation into the dorsal hippocampus. This produced four experimental diet groups: LFD-saline (*n* = 23; LFD-SAL), LFD-insulin (*n* = 23; LFD-INS), HFD-saline (*n* = 23; HFD-SAL), and HFD-insulin (*n* = 23; HFD-INS). Bilateral dorsal hippocampal cannulae (28-gauge, Alzet brain infusion kit; BioScientific Pty Ltd, Sydney, NSW; coordinates relative to bregma; AP−2.0mm, ML +/−1.8mm, DV−1.6mm from dura) were implanted and attached to osmotic mini-pumps (Alzet model 2004; BioScientific Pty Ltd, Sydney, NSW) infusing insulin or saline. Mice were anesthetized with isoflurane at an induction rate of 5% and a maintenance rate of 1–2% (Advanced Anesthesia Specialists, Gladesville, NSW) before being placed in a stereotactic frame (Kopf Instruments, Tujunga, CA). The mini pump infused Humulin N insulin (Eli Lilly and Co., Melrose Park, NSW) into the LFD-INS and HFD-INS groups at 2.64 μl per day at a concentration of 4 mU/μl for 28 days. In the LFD-SAL and HFD-SAL groups the mini pump infused saline at the same rate for 28 days.

Mice received a single post-surgery subcutaneous injection of analgesia (Ilium Ketoprofen 5 mg/kg; Provet, Cameron Park, NSW) and were single housed for 1 week (37 × 23 × 14 cm) to allow for monitoring of behavioral signs of discomfort, complications with wound healing, and any significant loss of body weight. During this week mice were gently handled to minimize the stress of behavioral testing. Mice that did not regain normal activity within 24 h of surgery were euthanized via cervical dislocation following anesthesia ( *n* = 7).

### Behavioral Testing

Spatial memory was assessed using a Morris water maze (MWM) and Y-maze (31–36). Behavioral testing started 1 week after surgery. On each day of testing, mice were transported to the experimental room 30 min prior to commencement of behavior tests. For both the MWM and Y-maze tests, the maze was located in the center of an experimental room and was surrounded by a curtain. Behavior throughout testing was recorded to computer via an overhead camera for later scoring using HVS Image Software.

#### Morris Water Maze

**Figure 1:**
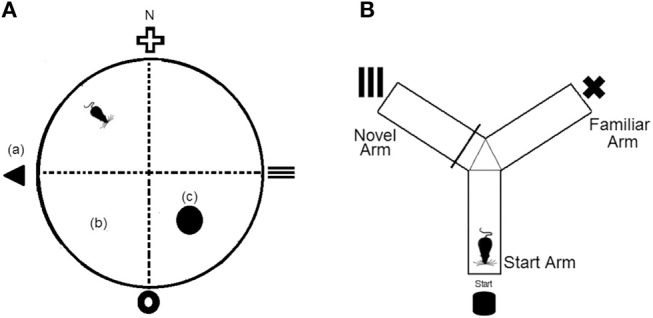
**(A)** Representation of the Morris Water Maze (MWM) apparatus, which was divided virtually into four quadrants. (a), fixed distal cues; (b), virtual maze quadrants; (c), platform. *N* = north **(B)** Representation of the Y-maze apparatus which consists of three equal arms and three separate fixed distal cues.

The Morris Water Maze was conducted in a round tank (120 cm diameter × 60 cm depth) that was filled with 30 cm of water kept at 24 ± 2°C. The water was rendered opaque by addition of non-toxic tempera powder (Staples Australia Pty Ltd, Mascot, NSW). Distal cues (15 cm wide × 15 cm tall) were fixed to the curtain surrounding the pool and located at each of the four cardinal points and platform (10 cm diameter) was located in the SE quadrant of the maze (Figure 1A). Daily means were calculated for escape latency times of each mouse and experimental group over the five training days.

On Day 1 each mouse underwent two familiarization trials where the platform was colored with black and white stripes and was raised 5 mm above the water level. The mouse was provided 60 s to find the platform and if it did not do so in this time was gently guided onto the platform. The mouse remained on the platform for 15 s before being removed to its cage for a 5 min inter-trial interval (ITI). Over the following three consecutive days (Day 2 to Day 4), each mouse received four acquisition trials per day. The platform was covered in white tape and submerged 5 mm below the surface. On each trial, a mouse was placed into the tank from one of four start positions and allowed 60 s to find the submerged platform. If a mouse did not find the platform in this time, it was guided to the platform location. The mouse remained on the platform for 15 s before being picked up and placed back into the tank at one of the other four start positions. This was continued until the mouse had been allowed to find the submerged platform from all four quadrants. On day 5, a 90 second probe trial, in which the platform was removed from the maze, was conducted to assess reference memory. The swim path, swim length, and time in target quadrant were all measured.

#### Y-Maze

The Y-maze consisted of three identical size arms (35 cm arm length × 10 cm arm height × 5 cm corridor width) that were connected to a center zone. A visual cue (5 × 5 cm) was presented at the end of each arm and each arm was assigned as either the start arm, familiar arm, or novel arm (Figure 1B). To assess long term memory, mice underwent a 10 min training trial where access to the novel arm was blocked off. Following a 1 h retention interval, mice were returned to the Y-maze and provided unrestricted access to all three arms for a 5 min test period. Time spent in the novel arm was recorded.

### Glucose Tolerance Test

A glucose tolerance test (GTT) was conducted at 18 days post-surgery in the second cohort of mice (*n* = 32). After overnight fasting, blood was collected in capillary tubes from a tail cut prior to intraperitoneal injection of glucose (1 g/kg body weight) and at 15 min post injection for insulin assay. Blood was sampled at 0, 15, 30, 60, and 120 min post injection for glucose concentrations using a glucometer (Accuchek Performa).

### Insulin Tolerance Test

An insulin tolerance test (ITT) was conducted at 25 days post-surgery in the second cohort of mice (*n* = 32). Mice had food removed and were received an intraperitoneal injection of insulin (1 U/kg body weight). Blood glucose levels were sampled at 0, 15, 30, 45, and 60 min post injection using a glucometer (Accuchek Performa).

### Plasma Leptin and Insulin

Plasma insulin and leptin were determined by ELISA performed in duplicate to the manufacturers recommendations. Insulin assays were performed on plasma taken during GTT, leptin was assessed in plasma taken at sacrifice (Crystal Chem).

### Real-Time PCR

RNA was extracted using Trireagent and RNA quality was determined via nanodrop Lite (Thermofisher Scientific) and converted to cDNA (Life technologies, SuperScript III First-Strand Synthesis). Real-Time PCR was performed with gene-specific TaqMan primers (Applied Biosystems), see Table 2. Reactions were performed in triplicate with the following cycling protocol: 360 s heat start at 95°C, 45 cycles of denaturation at 95°C for 25 s, annealing at 59°C for 30 s, and extension at 72°C for 20 s. Fluorescence detection was performed at 72°C. Relative expression was normalized to Ribosomal protein L32 (Rpl32) and β-actin (ACTB).

**Table 2:** UniGene and assay IDs for TaqMan gene expression analyses*.

**Gene**	**UniGene ID**	**Assay IDs**
IL-1B	316673–Mm.222830	Mm00434228_m1
TNF-a	256267–Mm.1293	Mm00443258_m1
IL-10	256068–Mm.874	Mm01288386_m1
IL-6	256136- Mm.1019	Mm00446190_m1
NFKB1	343411–Mm.256765	Mm00476361_m1
MCP-1	1066607–Mm.290320	Mm00441242_m1
BDNF	256365–Mm.1442	Mm04230607_s1
IGF-1	573947–Mm.268521	Mm00439560_m1

* *Obtained from ThermoFisher Scientific*.

### Statistical Analysis

Data were analyzed using Statistica 12.0 (Dell Software) and is presented as means with standard errors. Repeated measures (body weight and food intake) and 2-way between group ANOVAs (tissue weights, behavioral tests and gene expression) were followed by Tukey's honest significance difference (HSD) test for *post-hoc* analysis when a significant interaction effect was observed. Differences were accepted as statistically significant at *p* < 0.05

## Results

### Body Weight, Food Intake and Tissue Weights

**Figure 2:**
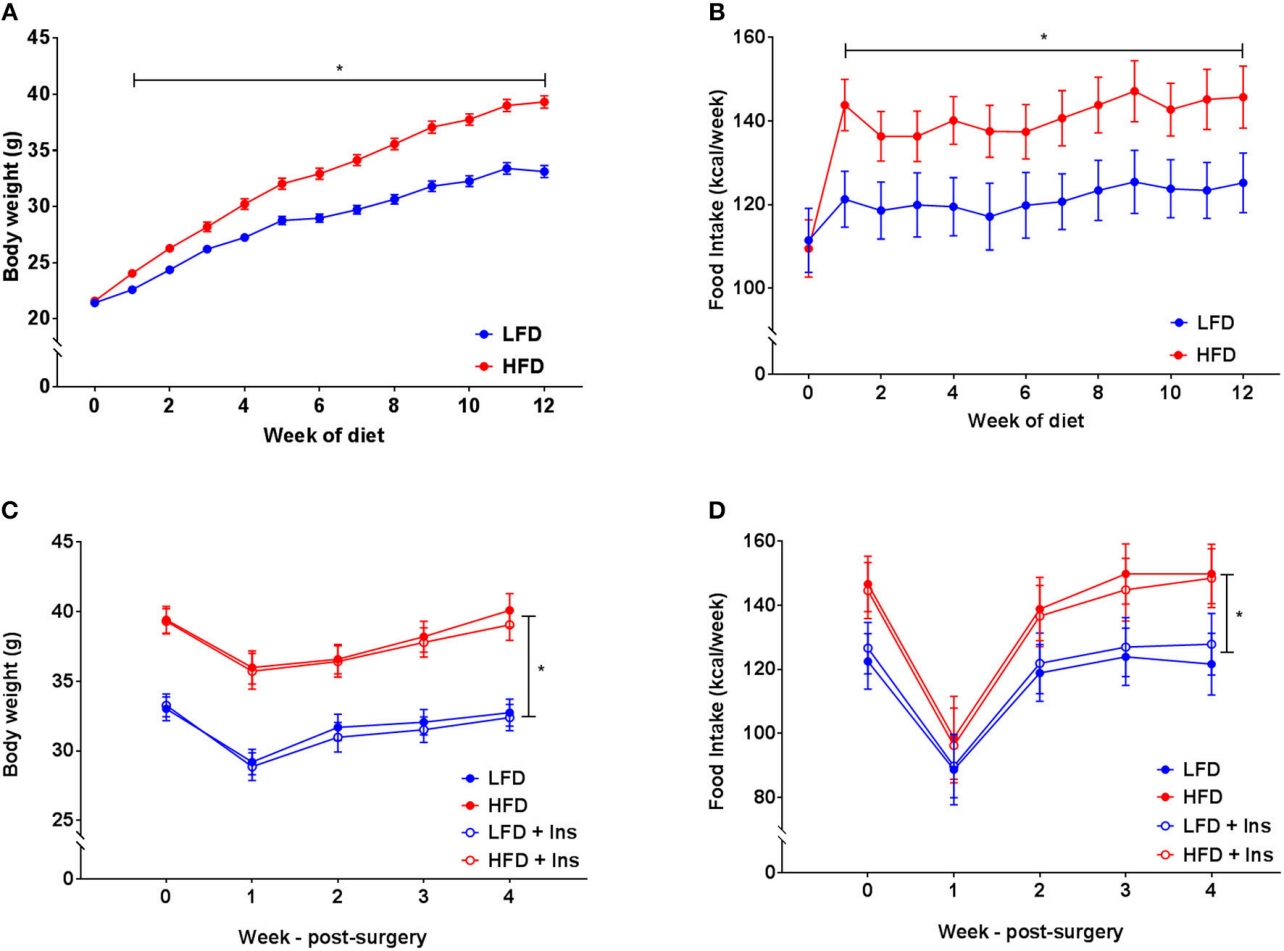
*Body weight and energy intake*. **(A)** After 1 week of high fat diet (HFD) feeding, mice had elevated body weight relative to low fat diet (LFD) controls, this was maintained throughout the study period. **(B)** Energy intake was elevated in HFD relative to LFD controls prior to surgery. **(C)** Body weights remained lower in LFD than HFD fed animals following surgery regardless of insulin infusion (+Ins). **(D)** Energy intake following surgery was greater in HFD than LFD fed animals. Values are expressed as mean ± SEM. *p < 0.05.

There was a significant interaction between diet and week on body weight (*p* < 0.05). Increased body weight was observed in HFD mice compared to the LFD group after two weeks on their respective diets (Figure 2A, *p* < 0.05). This was attributable to HFD mice consuming a greater amount of energy compared to mice in the LFD group (Figure 2B, main effect of diet, *p* < 0.05). All mice lost weight following surgeries, however, HFD mice still weighed more than LFD mice and this was unaffected by insulin infusion (Figure 2C, main effect of diet, *p* < 0.05). Overall energy intake following surgery was greater in HFD animals but not affected by insulin infusion (Figure 2D, main effect of diet, *p* < 0.05).

**Figure 3:**
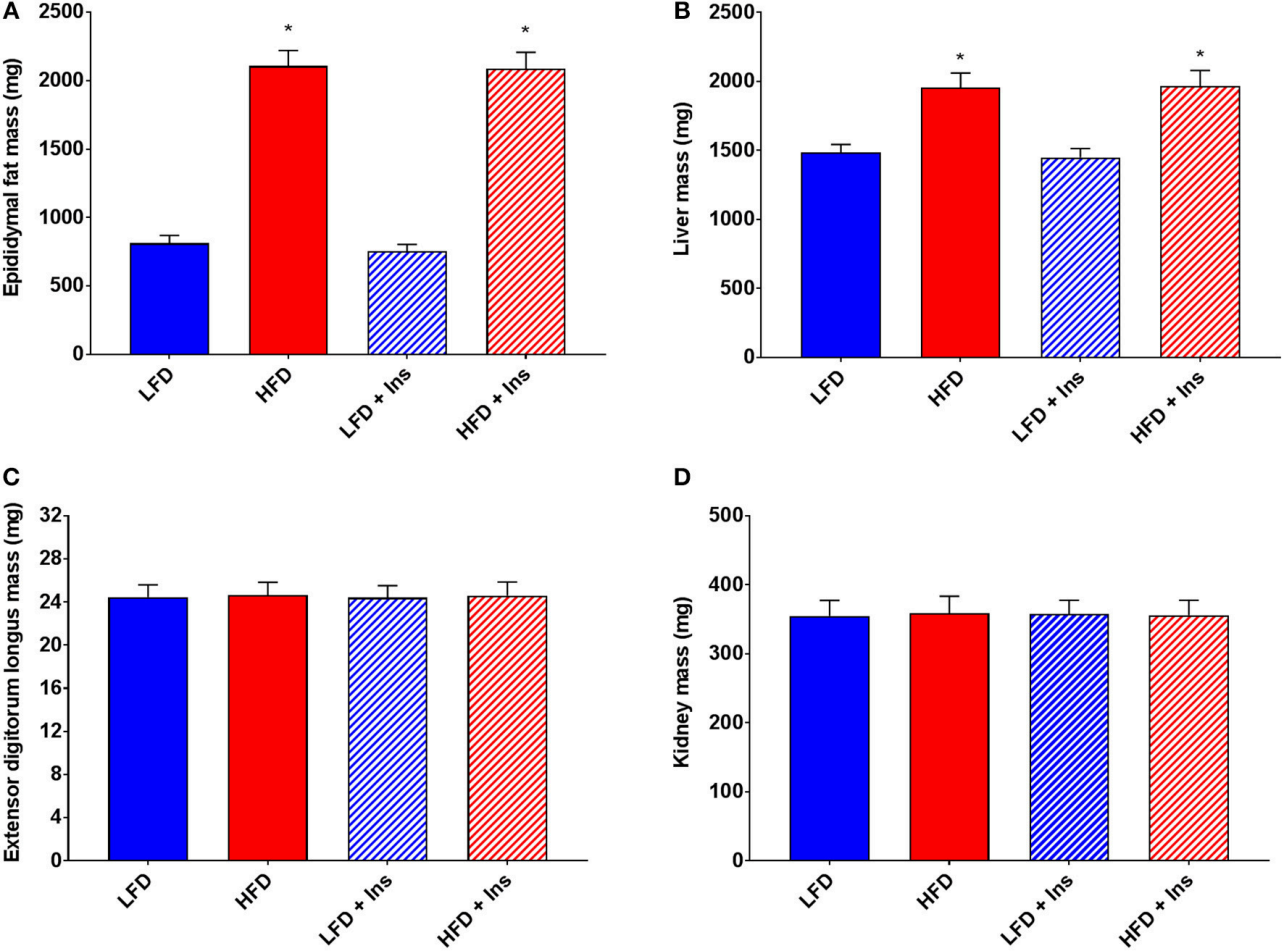
*Peripheral tissue weights*. **(A)** High fat diet (HFD) was associated with greater epididymal fat and **(B)** liver mass, but not **(C)** extensor digitorum longus muscle and **(D)** kidney mass compared with low fat diet (LFD) controls. Intrahippocampal insulin infusion (+Ins) did not affect any tissue weights. Values are expressed as mean + SEM. *p < 0.05.

Organ measurements were assessed to ensure intrahippocampal insulin had no significant effect on the peripheral physiology associated with HFD feeding. Increases in epididymal fat mass and liver mass were associated with both of the HFD groups compared to the LFD groups (Figures 3A,B, main effects of diet, *p* < 0.05). Central insulin infusion did not have an effect on these peripheral responses to diet (Figures 3A,B). No effects were observed in extensor digitorum longus muscle and kidney mass regardless of diet or infusion group (Figures 3C,D).

### Glucose Intolerance and Insulin Sensitivity

**Figure 4:**
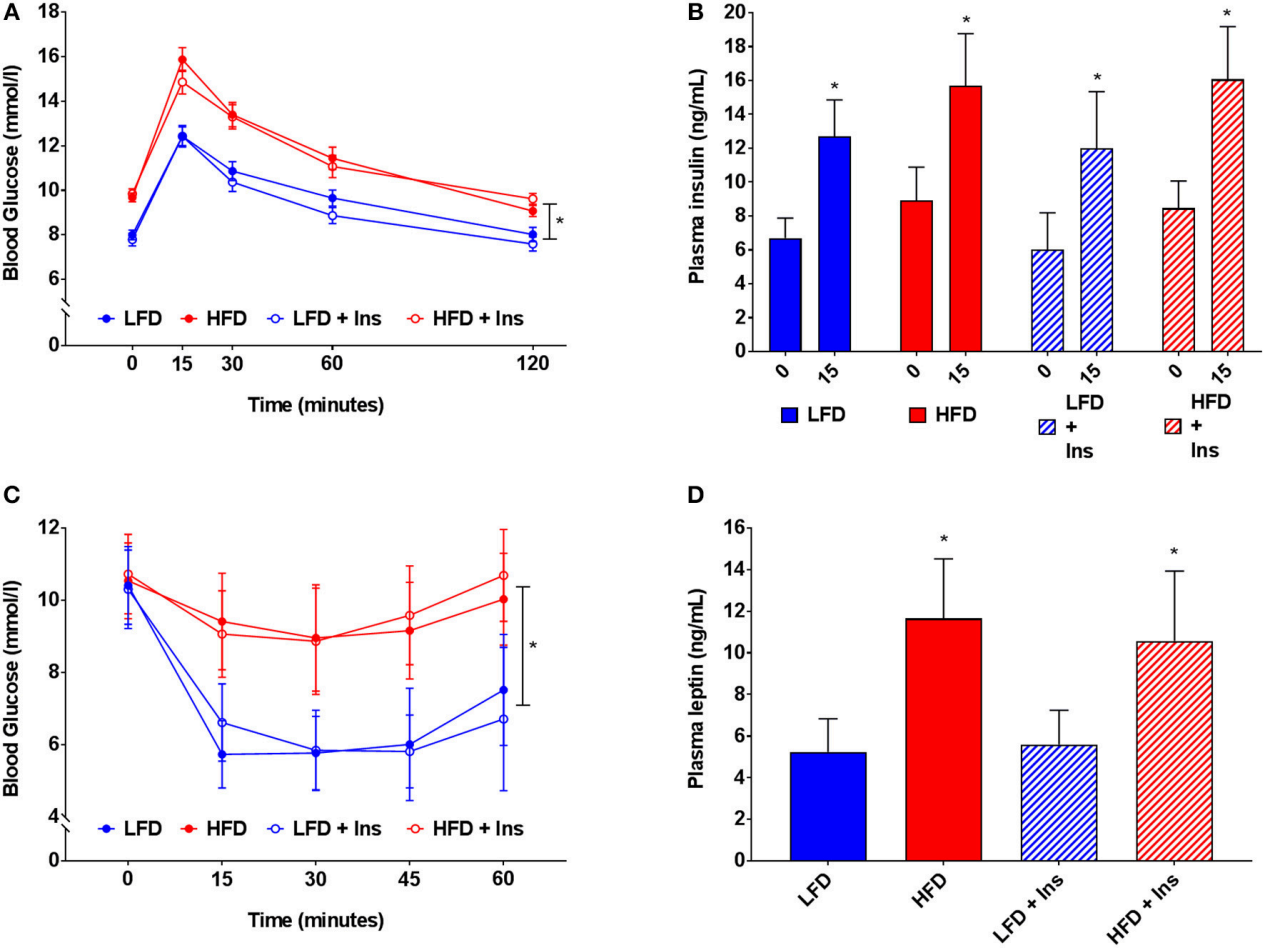
*Blood glucose, insulin and leptin*. **(A)** High fat diet (HFD) produced glucose intolerance compared to low fat diet (LFD) controls. **(B)** However, plasma insulin levels were not significantly different between HFD and LFD at baseline or 15 min after glucose injection. **(C)** HFD caused insulin insensitivity compared to LFD controls, with a smaller reduction in blood glucose after insulin injection. **(D)** HFD mice had higher plasma leptin levels than LFD controls. Glucose tolerance, insulin sensitivity, and plasma leptin were not influenced by intrahippocampal insulin infusion (+Ins). Values are expressed as mean ± SEM. *p < 0.05.

HFD caused glucose intolerance and insulin insensitivity (Figures 4A–C), with HFD mice demonstrating higher blood glucose in response to glucose injection (Figure 4A, main effect of diet, *p* < 0.05). However, plasma insulin levels were not significantly different between LFD and HFD mice, either at baseline or 15 min after glucose injection, but were elevated in all groups at 15 min relative to baseline (Figure 4B, main effect of time, *p* < 0.05). Following peripheral insulin injection mice fed HFD had a smaller reduction in blood glucose than those on LFD (Figure 4C, main effect of diet, *p* < 0.05). Glucose tolerance and insulin sensitivity were not influenced by intrahippocampal insulin infusion.

### Plasma Leptin

Plasma leptin reflected the diets mice were fed (see Figure 4D). HFD fed mice had higher plasma leptin levels than LFD fed mice (Figure 4D, main effect of diet, *p* < 0.05). Intrahippocampal insulin infusion had no effect on plasma leptin.

### Morris Water Maze

**Figure 5:**
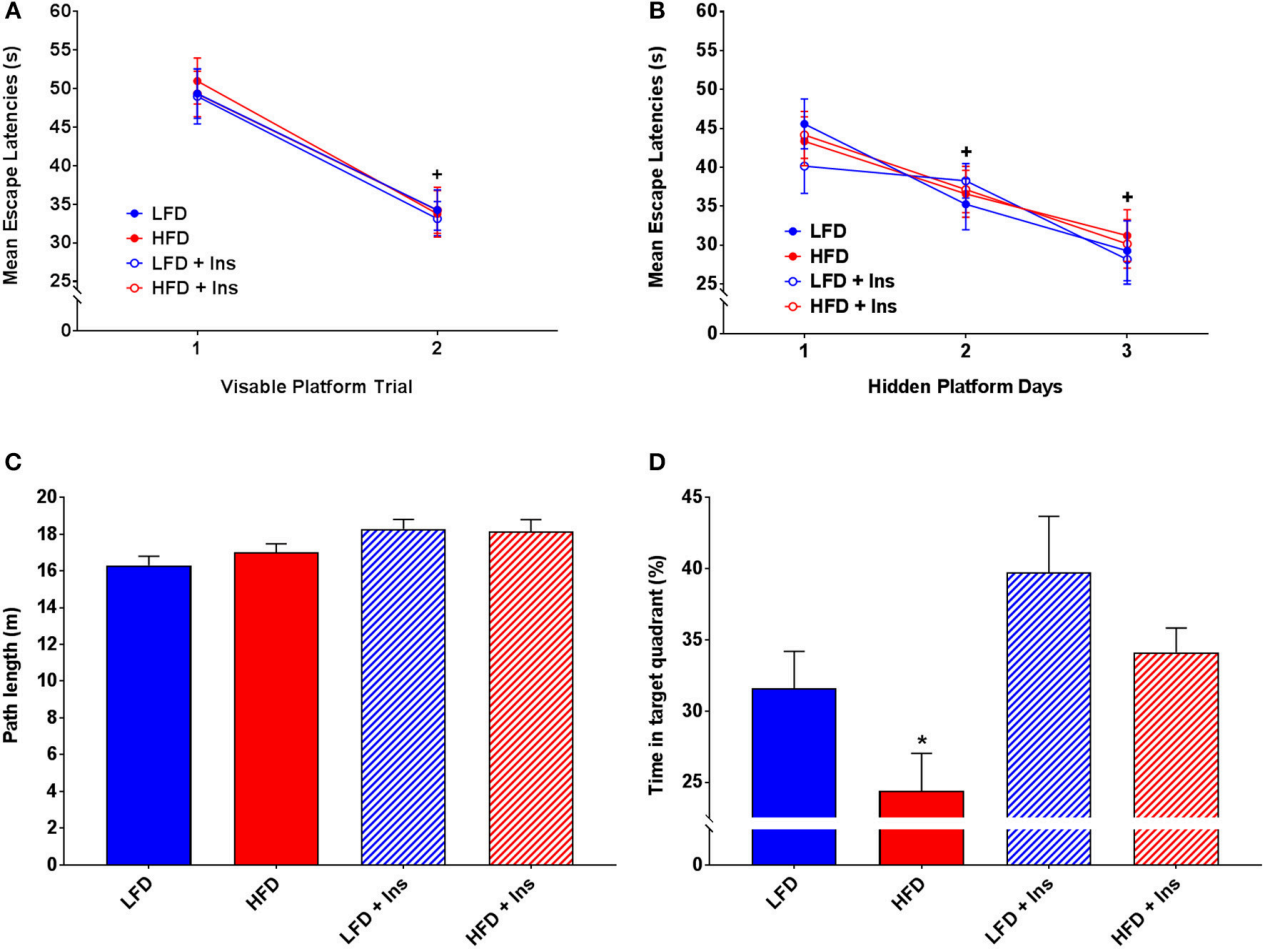
*MWM performance*. Mean escape latencies were collected for each trial day to assess performance over time. Time spent in the target quadrant was measured to assess hippocampal-dependent memory. **(A)** Mean escape latencies did not differ between groups on Day 1 of visible platform training. **(B)** All groups reached the platform faster across the hidden platform days. **(C)** All groups swam similar path lengths during the probe trial. **(D)** High fat diet (HFD) mice spent less time in the target quadrant compared with low fat diet (LFD) controls. Insulin infusion (+Ins) improved performance in both dietary groups compared to saline infused controls, with insulin infused mice spending more time in the target quadrant. Values are expressed as mean ± SEM. * = interaction effect, *p* < 0.05. ^+^ = main effect of time, *p* < 0.05.

All groups demonstrated similar escape latencies on Day 1 and Day 2 in the transition from visible to hidden platform training (Figures 5A,B). All groups began to reach the platform faster over the day 1 visible platform trials (Figure 5A; main effect of time, *p* < 0.05) and the hidden platform training days (Figure 5B; main effect of time, *p* < 0.05), indicating that there were no learning performance impairments across the groups.

All groups swam similar path lengths (Figure 5C), indicating that any differences observed were not due to sensorimotor or motivational deficits. There was a significant interaction effect on time in the target quadrant (*p* < 0.05), HFD saline mice spent less time in the target quadrant compared with LFD mice (Figure 5D; *p* < 0.05). Insulin infusion improved their performance, with mice in both dietary conditions spending more time in the target quadrant relative to HFD saline treated mice (Figure 5D; *p* < 0.05). In summary, HFD saline mice spent a smaller percentage of time in the target quadrant compared to LFD mice. Insulin infused mice, compared with saline infused mice, spent a greater percentage of time in the target quadrant (main effect of drug infusion *p* < 0.05).

### Y-Maze

**Figure 6:**
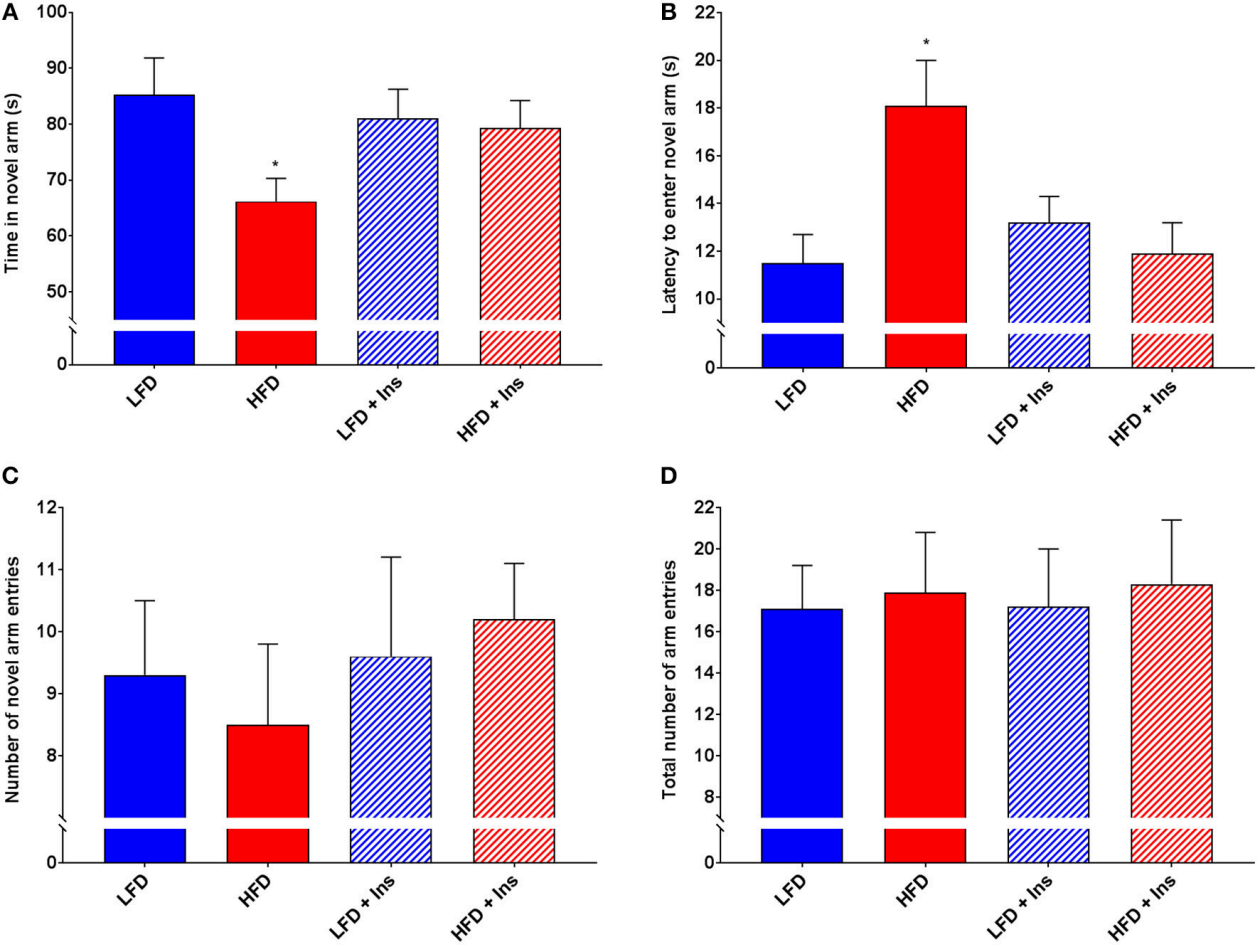
*Y-Maze performance*. Total arm entries were recorded to compare physical abilities. Novel arm entries, time spent in the novel arm, and latency to reach the novel arm were recorded as measures of hippocampal-dependent memory. **(A)** High fat diet (HFD) mice spent less time exploring the novel compared to low fat diet (LFD) controls. HFD intrahippocampal insulin infused (HFD+Ins) mice spent more time in the novel arm than HFD saline mice. **(B)** HFD mice took longer to initially enter the novel arm compared to LFD controls. HFD+Ins mice reached the novel arm faster than HFD saline mice. **(C)** Total novel arm and **(D)** Total arm entries did not differ between groups. Values are expressed as mean + SEM. *p < 0.05.

Interaction effects were observed for Time in the novel arm (Figure 6A, *p* < 0.05) and Latency to enter the novel arm (Figure 6B, *p* < 0.05). HFD saline mice spent less of their time exploring the novel arm (Figure 6A; *p* < 0.05) and took a longer time to reach the novel arm (Figure 6B; *p* < 0.05) compared to LFD mice. This was reversed by insulin infusion, with HFD insulin infused mice spending more time in the novel arm (Figure 6A; *p* < 0.05) and reaching it faster (Figure 6B; *p* < 0.05) compared to HFD saline infused mice. Despite this, HFD mice did not enter the novel arm of the maze fewer times than LFD mice (Figure 6C). There was also no significant difference in the number of total arm entries between any of the groups (Figure 6D), indicating that HFD mice were not physically impaired in comparison to LFD mice and that insulin infusion did not have an effect on the motivation of mice to explore the maze. Overall, HFD insulin infused mice reached the novel arm at times and for durations comparable to LFD mice.

### Hippocampal Inflammation

**Figure 7:**
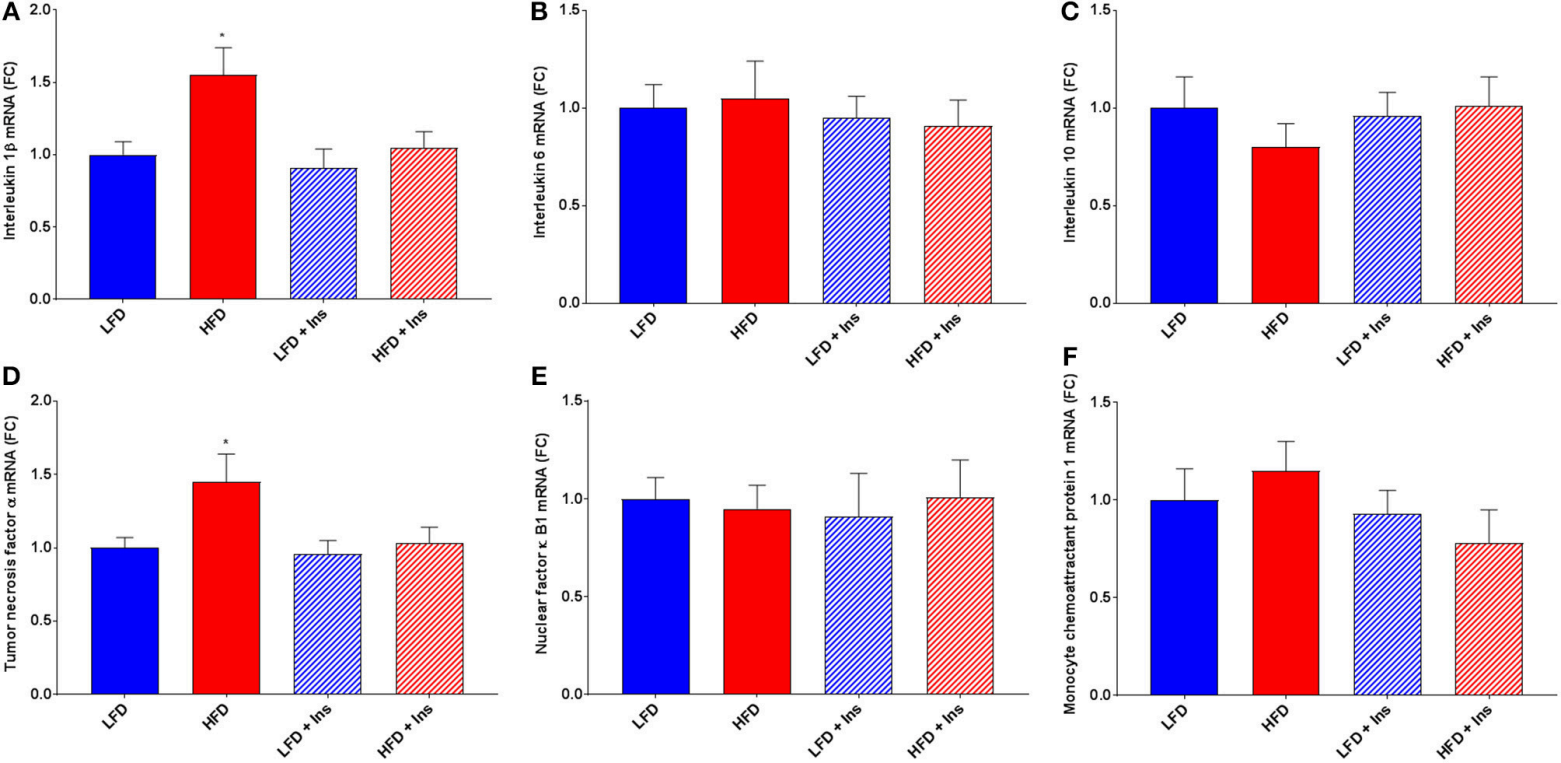
*Gene expression of cytokines*. Interleukin (IL)-1β, IL-6, IL-10, tumor necrosis factor (TNF)-α, nuclear factor (NF)κ-B1, and monocyte chemoattractant protein-1 (MCP-1) mRNA were measured in the hippocampus. **(A)** Expression of IL-1β mRNA was higher in high fat diet (HFD) mice compared to low fat diet (LFD) controls. HFD intrahippocampal insulin infused (HFD+Ins) mice expressed less IL-1β mRNA compared with HFD saline infused mice. Both LFD and HFD mice expressed similar levels of IL-6 **(B)** and IL-10 **(C)** mRNA; these levels were not affected by insulin infusion. **(D)** Expression of TNF-α mRNA was higher in HFD mice compared to LFD controls and HFD+Ins mice expressed less TNF-α compared to HFD saline infused mice. **(E)** Expression of NF-κB1 mRNA was comparable among all groups as was **(F)** MCP-1 expression. Values are expressed as mean + SEM. *p < 0.05.

**Figure 8:**
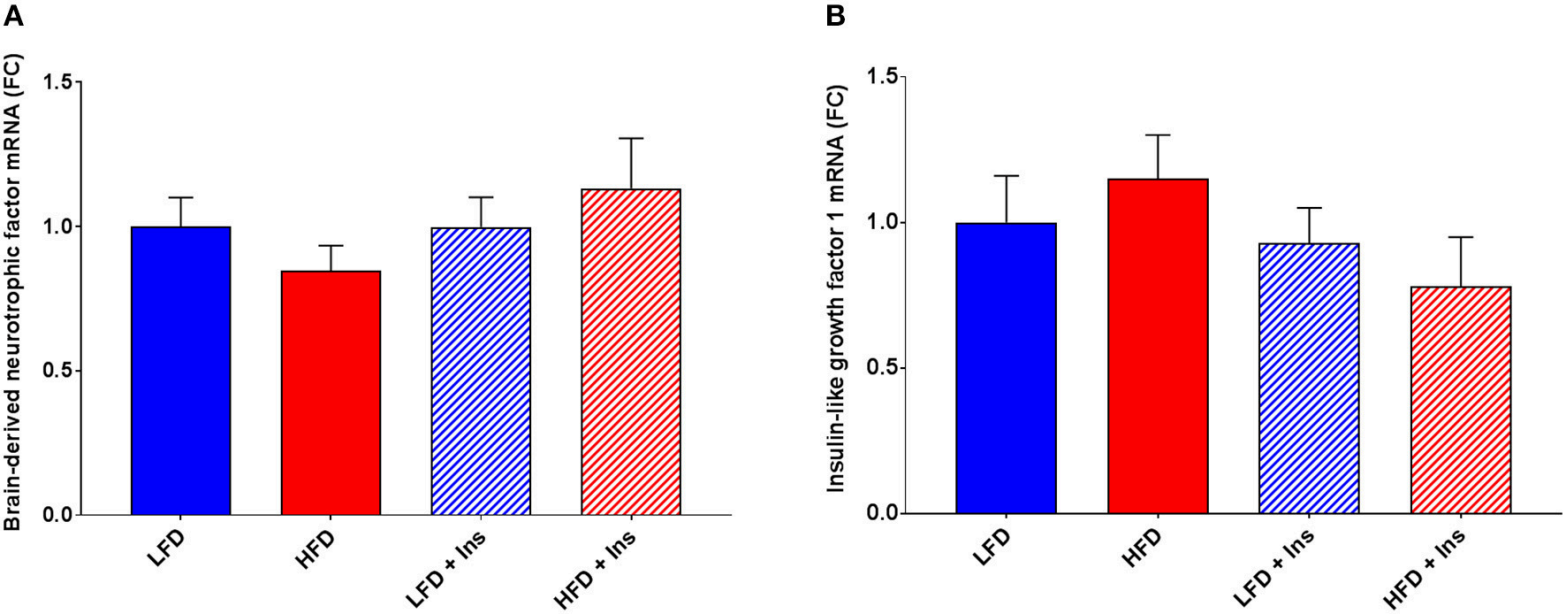
*Expression of growth factors*. Hippocampal brain-derived neurotrophic factor (BDNF) and insulin-like growth factor-1 (IGF-1) mRNA were measured in animals after sacrifice. Expression of both BDNF **(A)** and IGF-1 **(B)** were comparable among all groups. Values are expressed as mean + SEM.

The expression of the inflammatory markers IL-1β and TNF-α were higher in HFD mice compared to LFD mice (Figures 7A,D; *p* < 0.05), indicating an increase in inflammatory state in the hippocampus associated with HFD consumption. Chronic insulin infusion reduced IL-1β expression in the HFD insulin infused group compared to the HFD saline infused group to a level comparable to both LFD groups (Figure 7A; *p* < 0.05). Chronic insulin infusion also reduced TNF-α expression in HFD insulin infused mice compared to HFD saline infused mice, to a level comparable to both LFD groups (Figure 7D; *p* < 0.05). This indicates insulin can reduce neuroinflammation. Both LFD and HFD mice expressed similar levels of the cytokines IL-6, IL-10, nuclear factor k B1 (NFκ-B1), and monocyte chemoattractant protein 1 (MCP-1); these levels were not affected by insulin infusion (Figures 7B,C,E,F). Both LFD and HFD mice also expressed similar levels of Brain-derived neurotrophic factor (BDNF) and Insulin-like growth factor 1 (IGF-1); these were also not affected by insulin infusion (Figures 8A,B).

## Discussion

The present study examined the nature of hippocampal-dependent memory impairments in a mouse model of DIO and whether direct chronic insulin administration into the CNS could counteract these cognitive impairments mechanistically by reducing neuroinflammation. It examined the effect of DIO on hippocampal-dependent memory and neuroinflammation, as well as the effect of intrahippocampal insulin infusion on DIO-induced memory deficits and neuroinflammation. Consistent with previous research, mice consuming the HFD had significantly higher body weight than mice consuming LFD (7, 11, 15). The body weights were not influenced by insulin infusion, this is distinct from when insulin is infused into the third ventricle (37). It was further determined that HFD feeding resulted in glucose intolerance and peripheral insulin sensitivity.

Hippocampal-dependent memory performance was reduced following at least 13 weeks of diet access, with HFD mice spending significantly less time in the target quadrant in the MWM and making significantly fewer entries into the novel arm in the Y-maze when compared to all other groups. Intrahippocampal insulin infusion improved performance of HFD fed mice on both the MWM and Y-maze, with mice spending significantly greater time in the target quadrant in the MWM and making significantly more entries into the novel arm in the Y-maze when compared to saline infused HFD mice. Insulin infusion appeared to rescue HFD-induced deficits on the MWM and Y-maze, with HFD insulin and LFD saline mice demonstrating comparable performance at test on these tasks. These differences were not a consequence of the effect of DIO or insulin infusion on motivation, nor the swimming or locomotor abilities of mice, with the four experimental groups showing comparable escape latencies during MWM acquisition and total number of arm entries on the Y-maze. Thus, the present findings suggest that reduced performance of HFD mice on the hippocampal-dependent memory tasks is a consequence of HFD-induced impairments to hippocampal function, as reported previously (7, 11, 14, 38). Furthermore, these diet-induced deficits can be rescued with intrahippocampal insulin infusion.

The expression of neuroinflammatory markers IL-1β and TNF-α were significantly increased in mice consuming the HFD compared to LFD, however the concentration of IL-6, IL-10, NF-κB1, and MCP-1 were comparable across groups. Previous DIO animal models have also demonstrated an association between HFD consumption and elevated neuroinflammatory markers, such as TNF-α (15) and IL-1β (39), following demonstrated memory impairments on the T-maze (15) and Y-maze (39). Intrahippocampal insulin infusion attenuated the increased concentration of IL-1β and TNF-α, with HFD insulin mice having similar levels of both proinflammatory markers as LFD saline mice. There was no effect of insulin infusion on IL-1β and TNF-α concentration in mice consuming LFD. The present findings demonstrate some of the first evidence of the efficacy of chronic insulin infusion on attenuating hippocampal inflammation.

In a recent drug-induced animal model of inflammation, ICV administration of insulin decreased the expression of IL-1β and TNF-α in the hippocampus and improved performance in the MWM of young but not aged rats (30). Two key points of difference between our studies, is the method of insulin administration and animal model of inflammation used. While Adzovic et al. (30) demonstrated a therapeutic effect of insulin on inflammation in young rats with pharmacologically-induced neuroinflammation, they failed to demonstrate an effect in aging rats with, presumably, natural neuroinflammation. This might be attributable to age-related changes to insulin receptor sensitivity at the level of the hippocampus, with age a considerable risk factor for central insulin resistance (40). Central insulin resistance could reduce the likelihood of seeing an effect of ICV insulin administration on hippocampal-dependent tasks, given it's non-specific targeting. Insulin is primarily transported across the blood brain barrier (BBB) from cerebrospinal fluid via the saturable insulin receptor (41, 42). It is possible that the lack of therapeutic effect of ICV insulin in aging rats was due to age-related transport issues of insulin from the ventricles into the hippocampus. Our model of direct intrahippocampal insulin infusion bypasses issues of BBB sensitivity, holding greater translational value. Our results also show evidence of insulin's efficacy in a natural DIO-induced model of neuroinflammation, vs. Adzovic et al. (30) pharmacologically-induced LPS model. Whether insulin administration can attenuate cognitive impairments associated with age-related inflammation warrants further investigation, perhaps using more direct methods of administration in a natural model, to closer represent the underlying physiological mechanisms.

Our observation that intrahippocampal insulin infusion reduces DIO-induced neuroinflammation provides evidence of one potential mechanism via which insulin administration rescues hippocampal-dependent memory in a non-pharmacological obese mouse phenotype. Mechanisms may vary according to phenotype. Insulin's signaling pathway drives cellular responses (e.g., gene transcription), and protein translation and transport (43, 44). McNay et al. (25) showed that intrahippocampal delivery of insulin, and not IGF-1, enhanced spatial working memory via PI3K pathways, and that blockage of endogenous hippocampal insulin impaired cognitive performance. In support of this, we also saw no association between insulin infusion and IGF-1 expression. Together, these suggest that the observed results can be associated to direct insulin signaling effects.

While LFD insulin mice demonstrated improved performance on the MWM compared to LFD saline mice, performance was not improved on the Y-maze, which has been implicated to be dependent on both the hippocampus and non-hippocampal brain regions, such as the prefrontal cortex (45, 46). Lesions to the prefrontal cortex do not reduce MWM performance (47), suggesting that insulin administration might only be useful for particular tasks, perhaps those more heavily or specifically dependent on the hippocampus. This is important given the critical role the hippocampus plays in spatial and episodic memory (8, 11, 13, 48). Further investigations are required to determine the localized effects of insulin in the brain in order to identify the specific neural circuits involved in the insulin-mediated changes to cognitive performance.

One possible explanation for the differential performance of LFD insulin mice on the two hippocampal-dependent tasks is the differential energy requirements between the tasks (46). Neurons utilize glucose as their main energy source and this is not insulin dependent in normal circumstances (49). Under increased cognitive load which is required to perform a task, glucose turnover within neurons increases to support neuronal activity (50) and this may be dependent on insulin signaling. Increasing the supply of glucose to neurons during times of high cognitive demand, such as during learning (51) or memory retrieval (52), is recognized to increase cognitive performance (51, 52). Insulin is a mediator of glucose availability within the hippocampus via translocation of glucose transporter-4 (GLUT-4) to the neuron membrane (53), which facilitates the uptake of glucose from the extracellular fluid (31, 32, 54, 55).

The improvement in spatial memory observed in HFD-mice receiving intrahippocampal insulin infusion provides further evidence toward the growing body of literature advocating the use of insulin as a therapeutic agent for neurodegenerative disorders (56, 57). The present findings provide some of the first evidence demonstrating one potential mechanism of action for the improvements observed in humans, although other mechanisms should not be overlooked. In addition to the beneficial effects of insulin administration seen in obese humans (58), numerous studies in rodents (25, 27–29) and healthy, non-obese humans (27, 59, 60) have demonstrated a potential for CNS insulin administration as a cognitive enhancer. The present study provides some evidence to support this hypothesis, with improved performance on the MWM in LFD insulin mice.

The high density of insulin receptors in the hippocampus have been postulated to have regulatory effects on cognition by controlling synaptic density and plasticity (54, 55). Pharmacologically induced insulin resistance reduces hippocampal neuroplasticity, long term potentiation, and spatial memory performance in the MWM (61). HFDs and subsequent insulin resistance have also been demonstrated to alter hippocampal synaptic plasticity (17). To support a role of insulin, deletion of insulin receptor substrate 2 reduces hippocampal NMDA receptor dependent synaptic plasticity (62). Thus in non-obese, LFD fed, and non-insulin resistant subjects, insulin may act mechanistically at the hippocampus to regulate synaptic plasticity and cognition. However, neither diet nor insulin altered the expression of BDNF, a protein involved in multiple signaling pathways that also regulate synaptic plasticity and memory formation (63). It is possible that insulin and BDNF alter synaptic plasticity via different pathways. Additionally, the two common housekeeping genes used for PCR analysis, Rpl32 and ACTB, might not be most stable in this DIO model (64). Validating optimal housekeeping genes according to the animal model being used will be considered in future.

## Conclusion

The current experiment demonstrated that DIO in mice produced impaired performance on hippocampal-dependent memory tasks, and that these deficits could be rescued with chronic intrahippocampal insulin administration. It was additionally shown that DIO was associated with increased concentration of proinflammatory cytokines, and that these were reduced by chronic intrahippocampal insulin infusion. It is hypothesized that the decrease in proinflammatory cytokines in insulin-infused HFD-fed mice mediates the improved performance on the MWM and Y-maze observed in these animals. The chronic infusion of insulin in this study has great translational value when comparing to current progress being made with intranasal insulin administration to humans. Understanding how discrete regions and physiological mechanisms of the CNS are affected by the pathologies of obesity and how they respond to different treatment methods is critical, particularly considering the growing population of obese individuals, and the link between obesity and heightened dementia risk.

## Author Contributions

DB designed the experiments. JG, KA, CA, AS, and DB collected and analyzed the data. JG and DB wrote the manuscript. KA, CA, and AS reviewed and edited the manuscript.

### Conflict of Interest Statement

The authors declare that the research was conducted in the absence of any commercial or financial relationships that could be construed as a potential conflict of interest.
